# Primary Cutaneous CD4+ Small/Medium T-cell Lymphoproliferative Disorder in a Young Japanese Male Patient

**DOI:** 10.7759/cureus.82211

**Published:** 2025-04-13

**Authors:** Yuta Norimatsu, Mina Komuta, Yuichiro Hayashi, Kennosuke Karube, Makoto Sugaya

**Affiliations:** 1 Department of Dermatology, International University of Health and Welfare Ichikawa Hospital, Ichikawa, JPN; 2 Department of Dermatology, International University of Health and Welfare Narita Hospital, Narita, JPN; 3 Department of Diagnostic Pathology, International University of Health and Welfare Narita Hospital, Narita, JPN; 4 Department of Anatomic Pathology, International University of Health and Welfare Narita Hospital, Narita, JPN; 5 Department of Pathology and Laboratory Medicine, Nagoya University, Nagoya, JPN

**Keywords:** bcl6, cutaneous pseudolymphoma, nuclear staining for nuclear factor of activated t-cells c1 (nfatc1), primary cutaneous cd4+ small/medium t-cell lymphoproliferative disorder (pcsm-lpd), programmed death 1 (pd-1), t-cell receptor gene rearrangement

## Abstract

A 34-year-old man became aware of an erythematous nodule on the left nasal wing. He was treated with topical steroids and oral antibacterial agents at his local doctor, but his condition did not improve, and he was referred to our hospital. A skin biopsy revealed diffuse cellular infiltration through the dermis. No epidermotropism was seen. The major infiltrate was small to medium-sized lymphoid cells. The number of CD3+ cells was almost the same as that of CD20+ cells, while CD4+ cells were dominant over CD8+ cells. Atypical lymphocytes were positive for BCL6 and PD-1. Polymerase chain reaction (PCR) analysis of immunoglobulin heavy chain and T-cell receptor gene rearrangements on paraffin-embedded tissue sections revealed a clonal expansion of T-cells. The patient was diagnosed as having primary cutaneous CD4+ small/medium T-cell lymphoproliferative disorder (PCSM-LPD) and treated with fludroxycortide tape. The red nodule completely disappeared after three months. Nuclear staining for nuclear factor of activated T-cells c1 (NFATc1), which had been suggested to be useful in distinguishing PCSM-LPD from pseudolymphoma, was negative in our case.

Our case was considered to be typical of PCSM-LPD among existing reports of PCSM-LPD from Japan, except for the young age of the patient. Our case suggested that young cases with PCSM-LPD may have been misdiagnosed with cutaneous pseudolymphoma (CPL), which may be one of the reasons why this type of lymphoproliferative disorder has been reported to occur in elderly people.

## Introduction

Primary cutaneous CD4+ small/medium T-cell lymphoproliferative disorder (PCSM-LPD) is synonymous with what used to be called primary cutaneous CD4-positive small- and medium-sized T-cell lymphoma, which has recently been renamed [1]. PCSM-LPD is characterized by nodules or plaques on the face, neck, and upper body [1]. Histology shows nodular or diffuse infiltration of CD4+ small to medium-sized T cells. The tumor cells are positive for follicular helper T-cell markers such as PD-1, BCL6, and CXCL13. The proliferation rate assessed by Ki-67 positivity is low, ranging from less than 5% to a maximum of 20% [1-5]. The male-to-female ratio of PCSM-LPD European patients was 1:1, and 91% of patients were said to be rash-free and mildly relieved within a median of 63 months. The median age of the disease onset in Europe is 53 years. The remaining 9% of patients survive with residual skin symptoms. PCSM-LPD often resolves spontaneously after skin biopsy but may be treated with topical steroids, surgery, or radiation therapy [6]. In the Japanese report, the male-to-female ratio of PCSM-LPD was 11:14 [7]. The median age was 61.5 years, older than that of Europeans. It was relatively rare, occurring in 25 (1.4%) of 1733 patients with cutaneous lymphoma diagnosed between 2007 and 2011 [7]. Here, we report a young patient with PCSM-LPD, who was successfully treated with occlusive topical steroid.

## Case presentation

**Figure 1:**
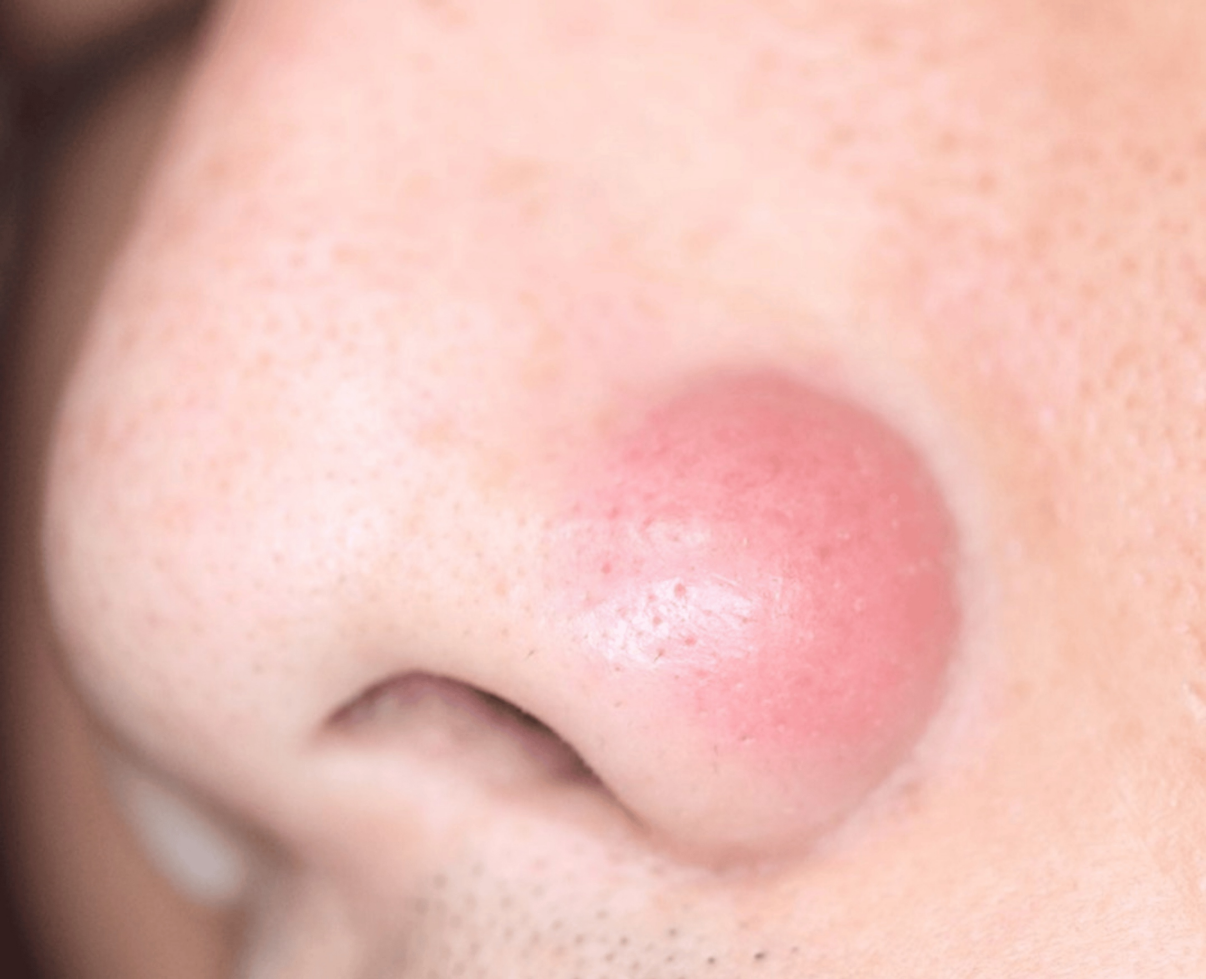
Red nodule on the left wing of the nose at the first visit.

A 34-year-old man was referred to our hospital with a suspicion of cutaneous lymphoma. The patient noticed a red nodule on the left nasal wing two months before. He visited his local doctor due to its gradual increase in size. He was treated with faropenem, topical bacitracin/fradiomycin sulfate, topical ketoconazole, and topical hydrocortisone butyrate, which were all ineffective. The nodule decreased in size while the patient took oral betamethasone, but it flared up when he stopped the drug. When the patient visited us, an erythematous nodule without ulceration was located on the left nasal wing (Figure 1).

**Figure 2:**
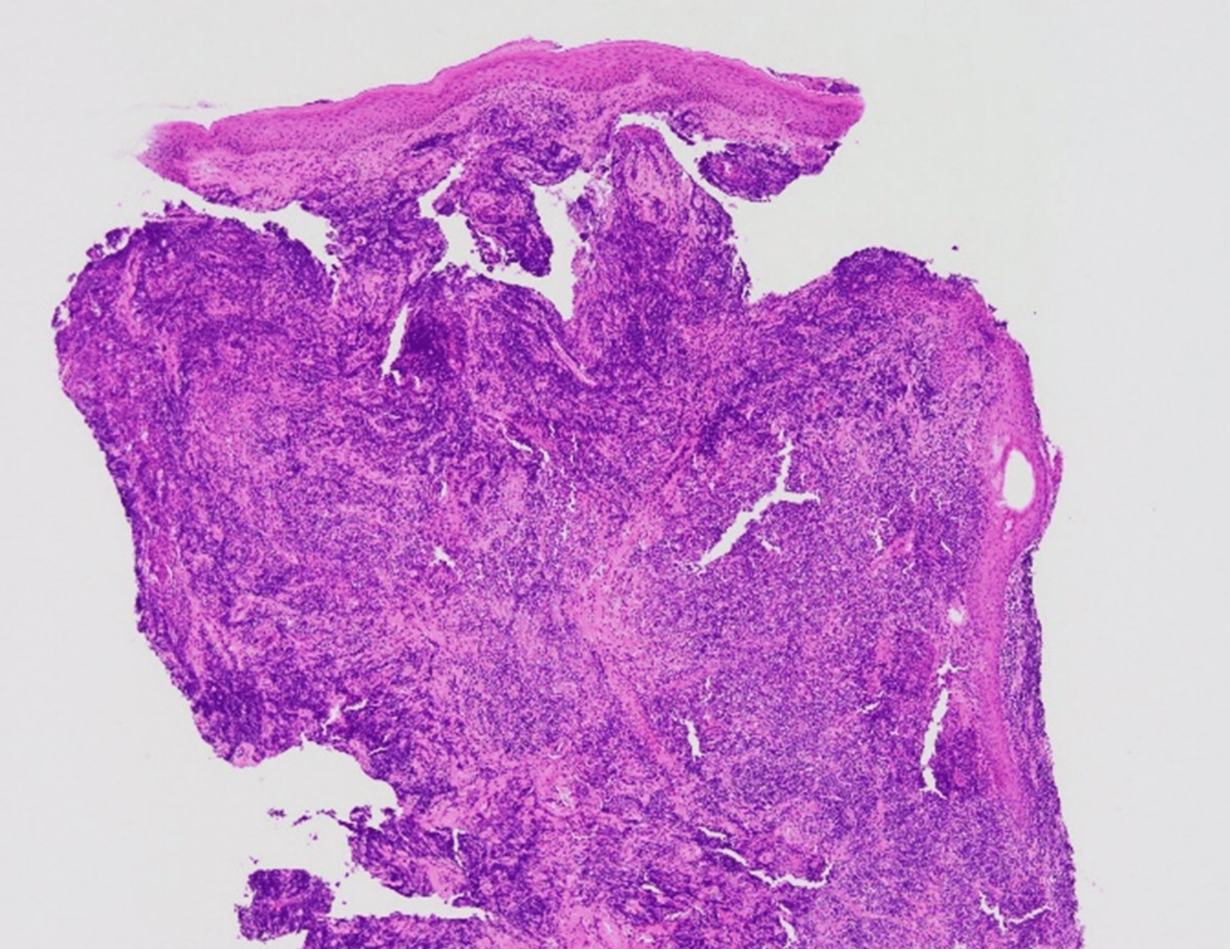
Hematoxylin and eosin stain shows significant cellular infiltration of the dermis (x40).

The skin biopsy specimen showed diffuse cellular infiltration through the dermis (Figure 2).

**Figure 3:**
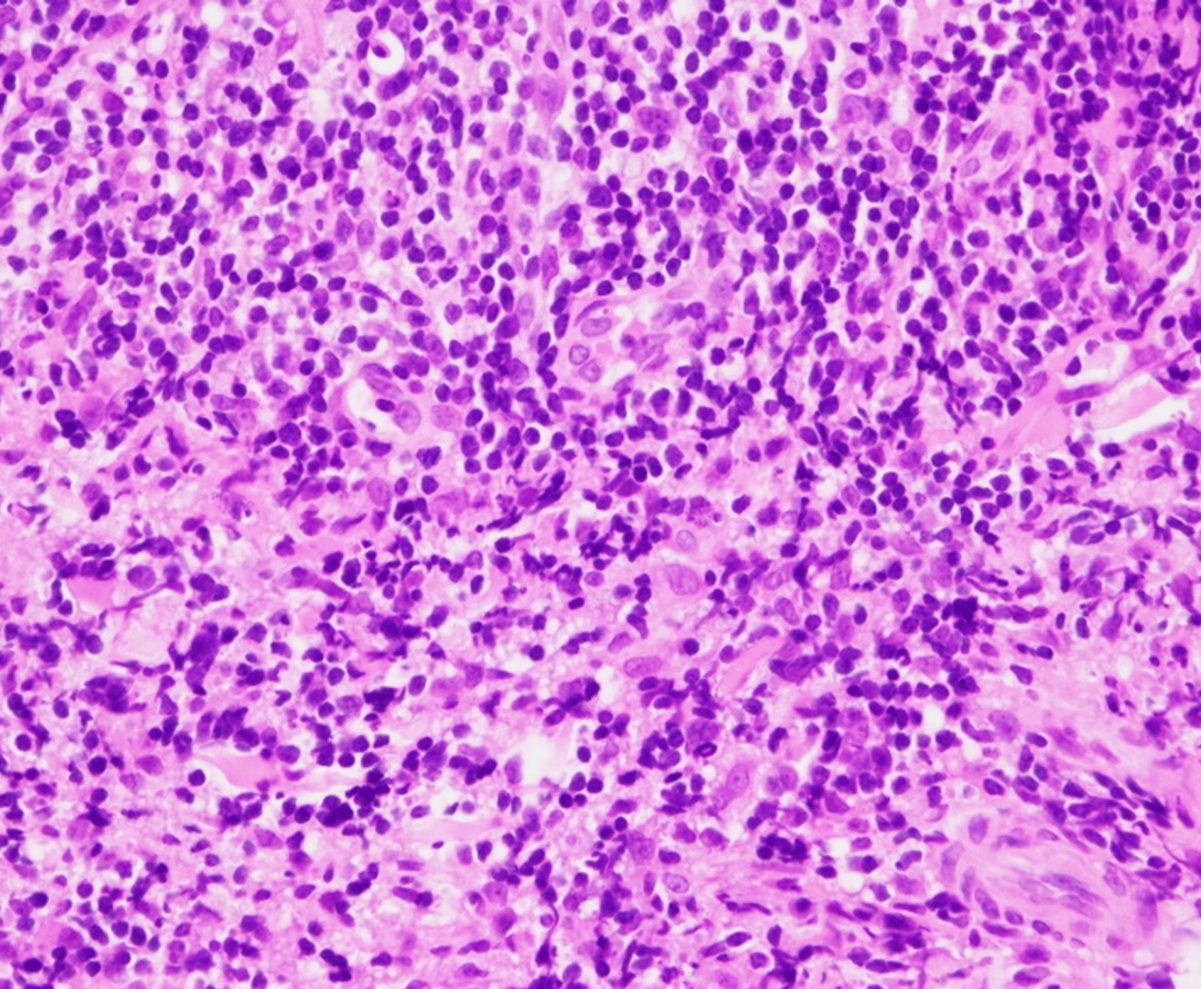
Cells infiltrating the dermis in hematoxylin and eosin stain are small to medium-sized lymphoid cells (x400).

No epidermotropism was seen. The major infiltrate was small to medium-sized lymphoid cells (Figure 3).

**Figure 4:**
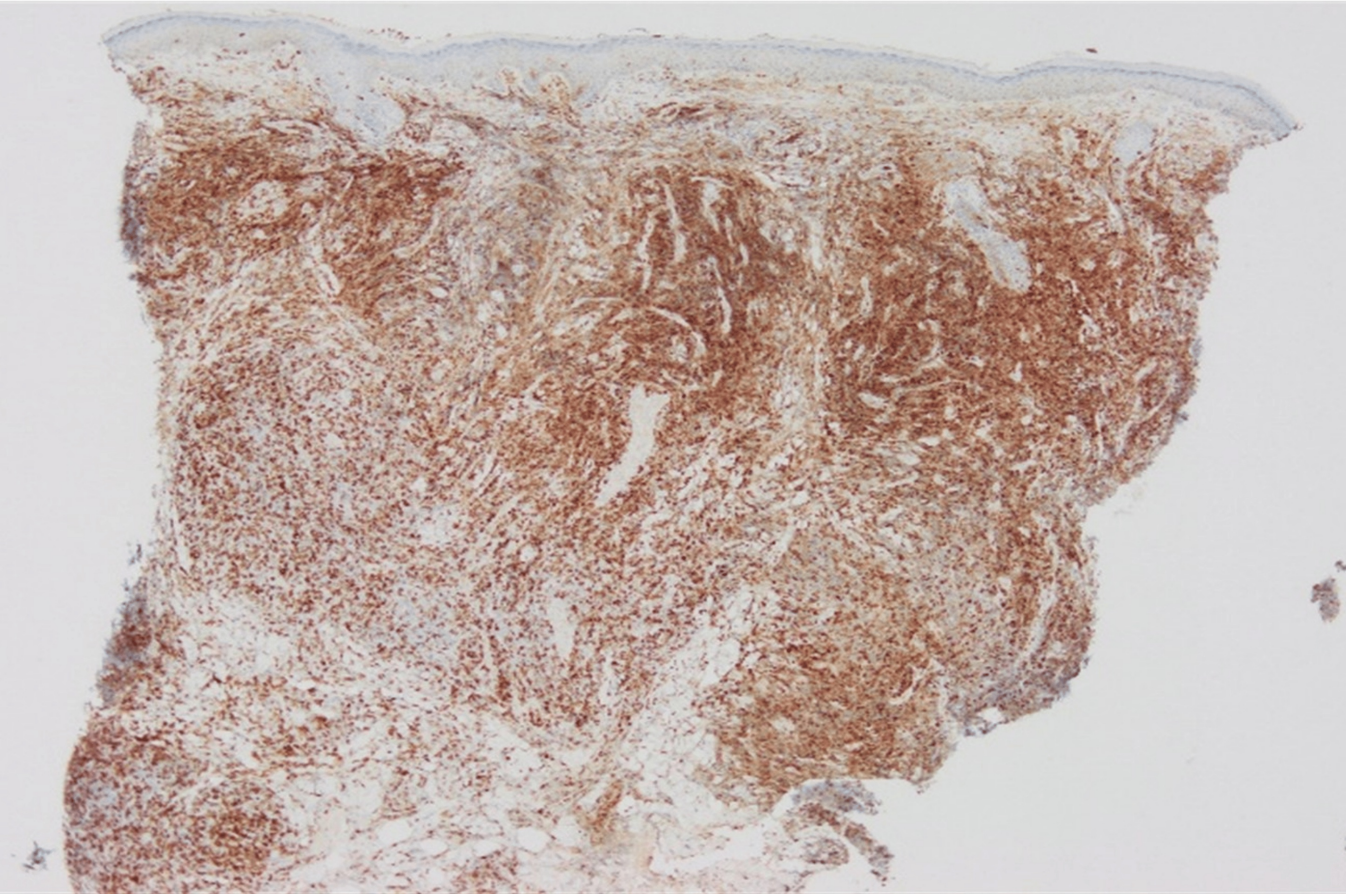
Immunohistological staining for CD3.

**Figure 5:**
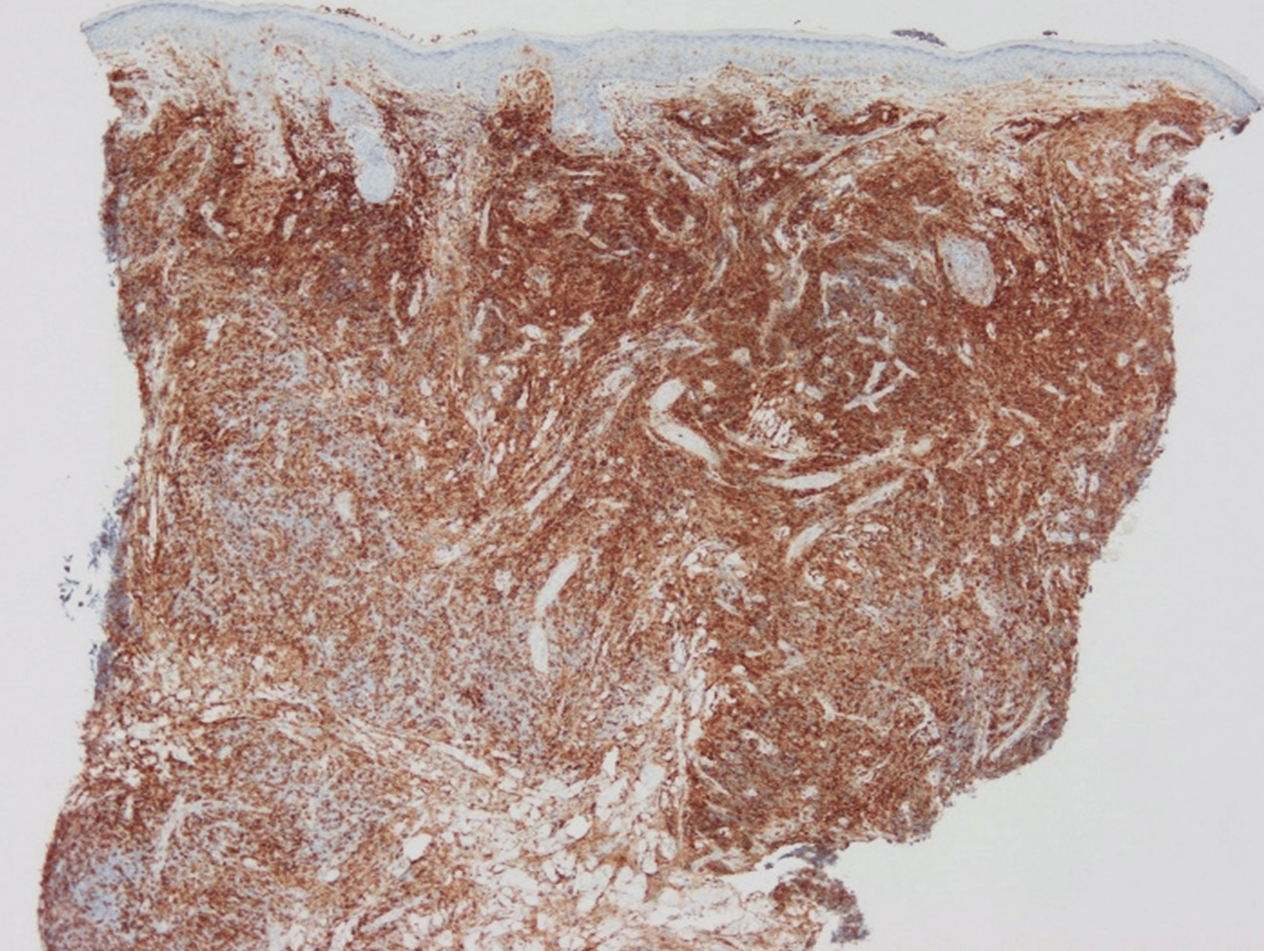
Immunohistological staining for CD4.

**Figure 6:**
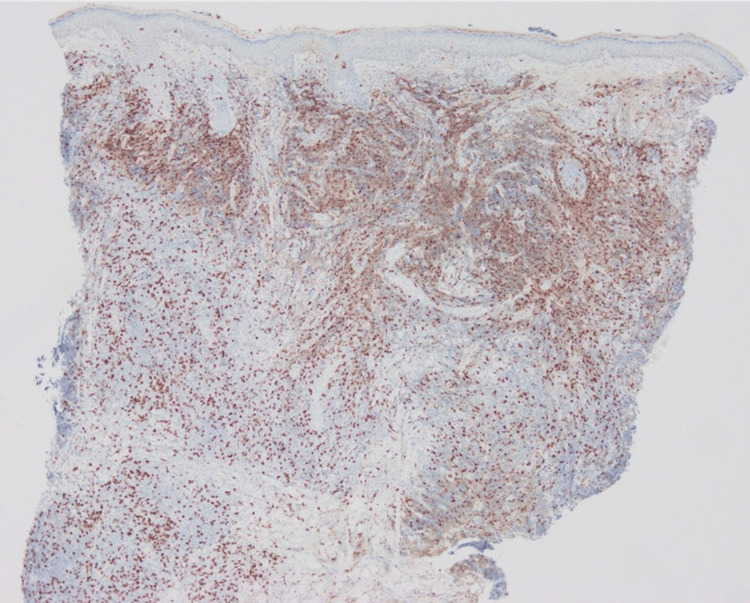
Immunohistological staining for CD8.

**Figure 7:**
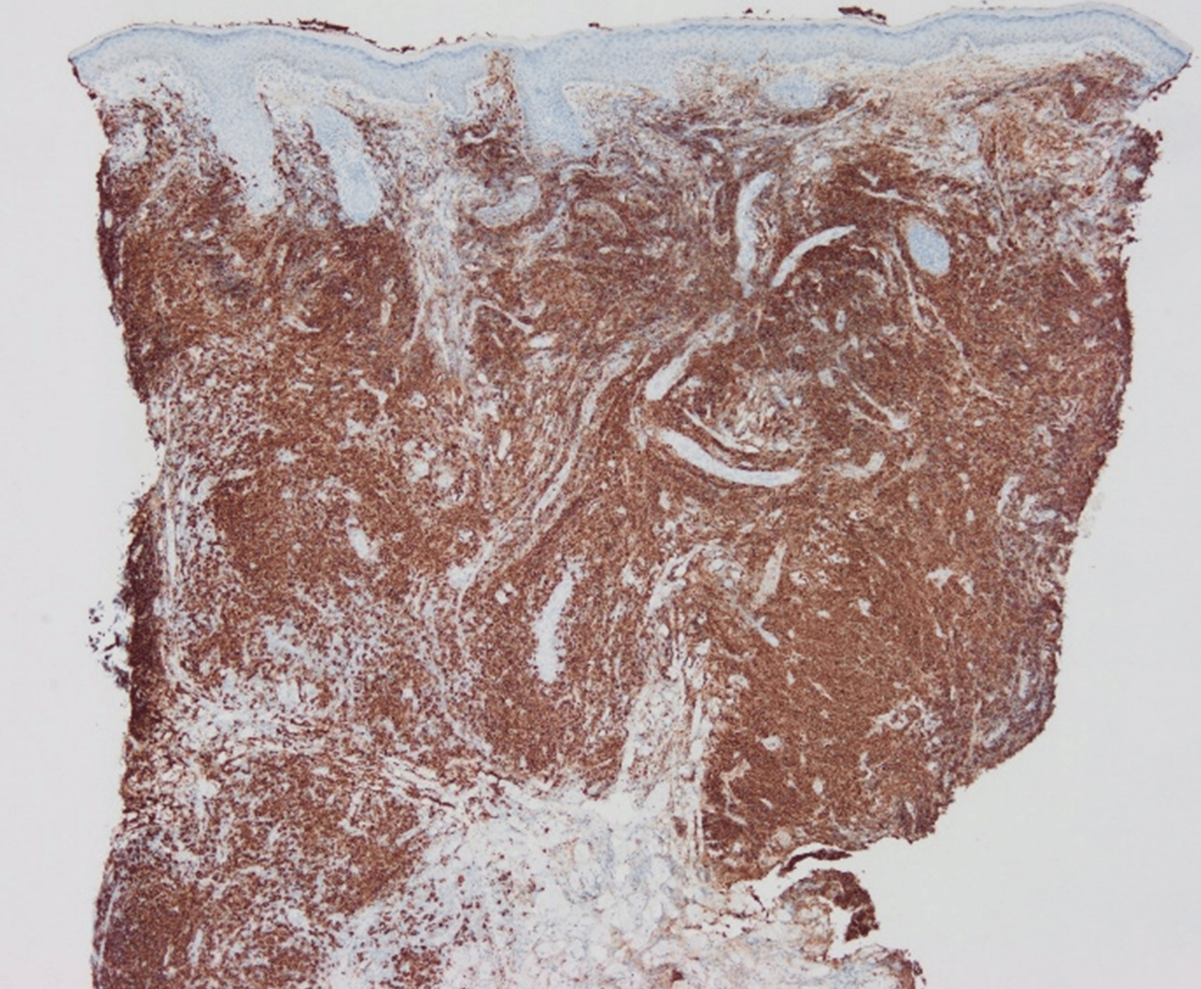
Immunohistological staining for CD20.

The number of CD3+ cells was almost the same as that of CD20+ cells, while CD4+ cells were dominant over CD8+ cells (Figures 4-7).

**Figure 8:**
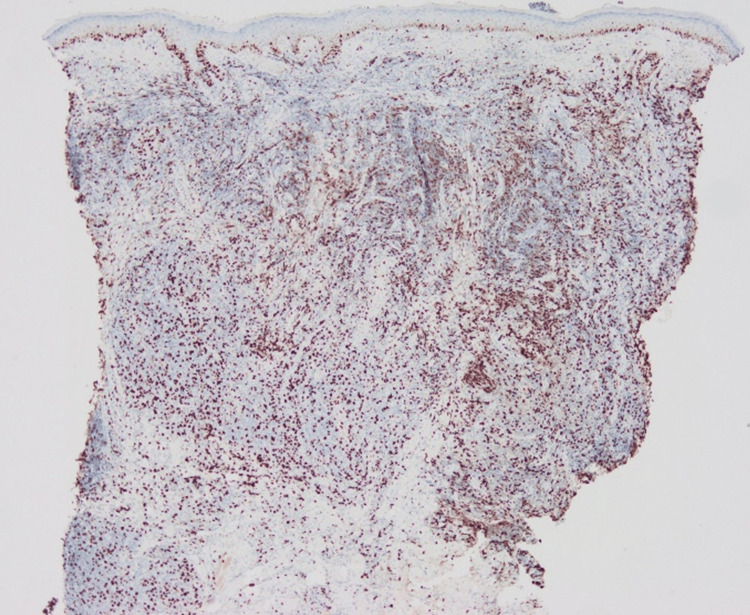
Immunohistological staining for Ki-67.

The ratio of Ki-67+ cells was about 20% (Figure 8).

**Figure 9:**
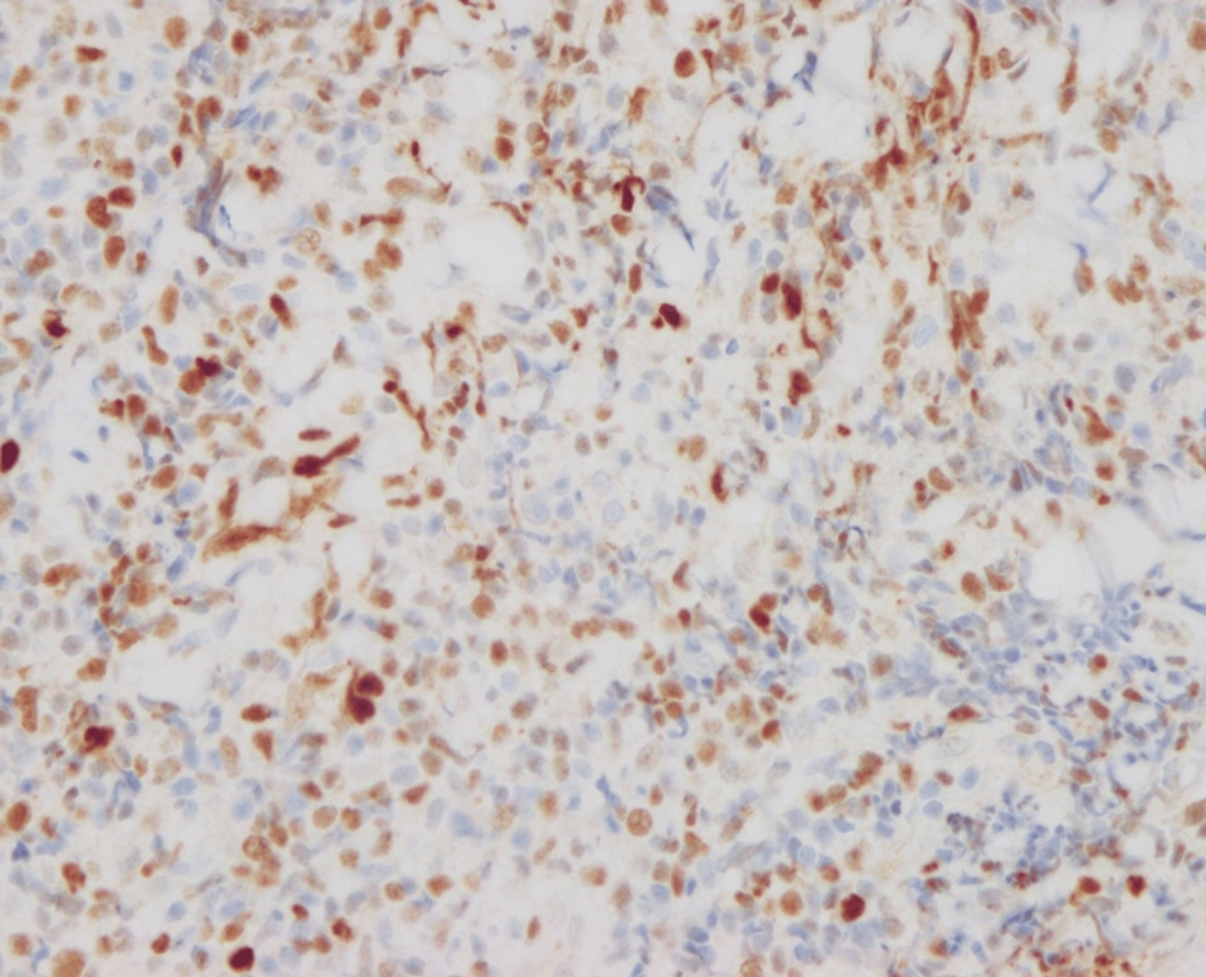
Immunohistological staining for BCL6.

**Figure 10:**
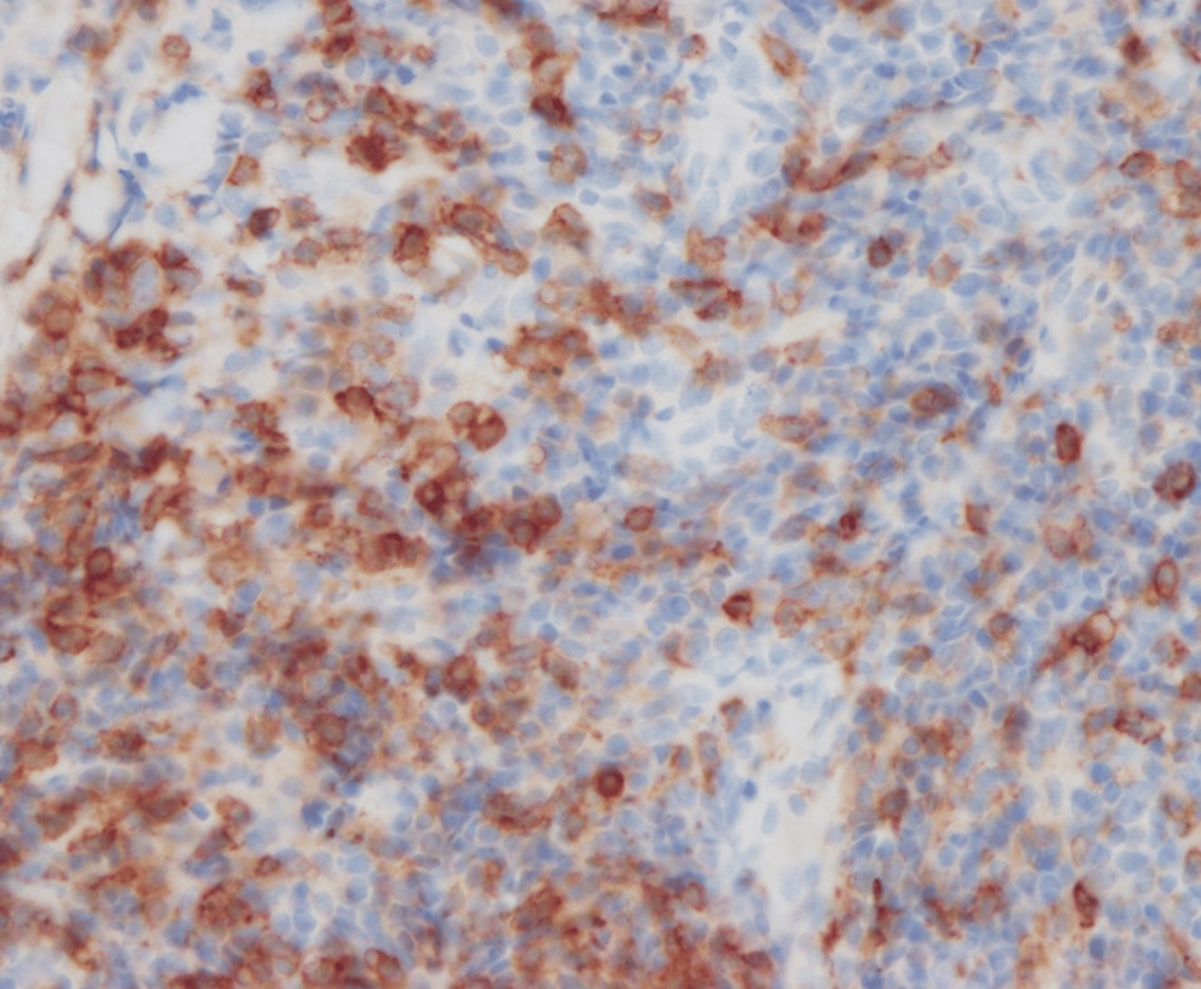
Immunohistological staining for PD-1.

Atypical lymphocytes were positive for BCL6 and PD-1 (Figures 9, 10).

**Figure 11:**
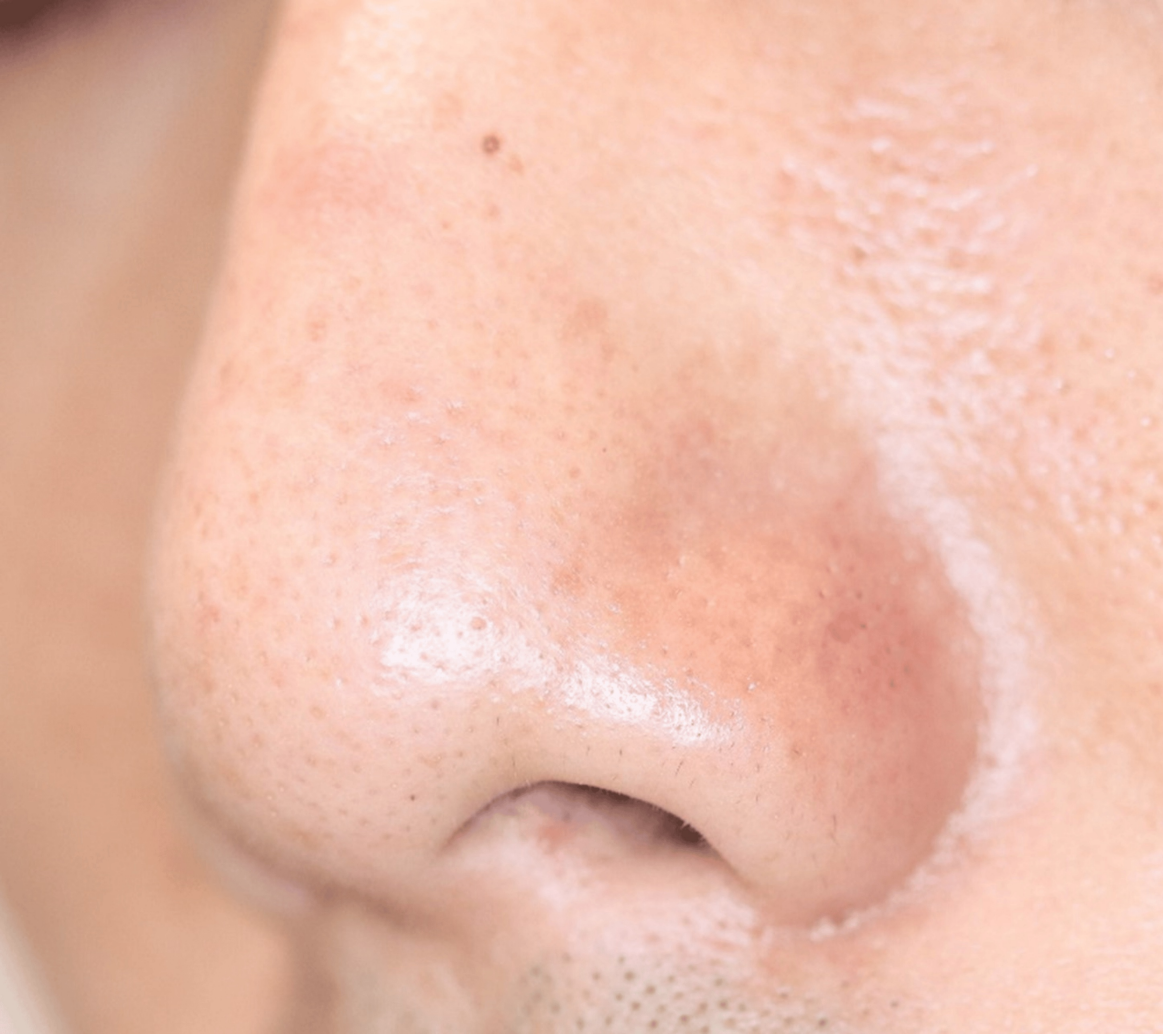
Red nodule on left wing of nose completely disappeared after three months.

Polymerase chain reaction (PCR) analysis of immunoglobulin heavy chain and T-cell receptor gene rearrangements on paraffin-embedded tissue sections revealed a clonal expansion of T-cells. CT scanning showed no extracutaneous lesions. The patient was diagnosed as PCSM-LPD and treated with fludroxycortide tape. The red nodule completely disappeared after three months (Figure 11).

## Discussion

**Figure 12:**
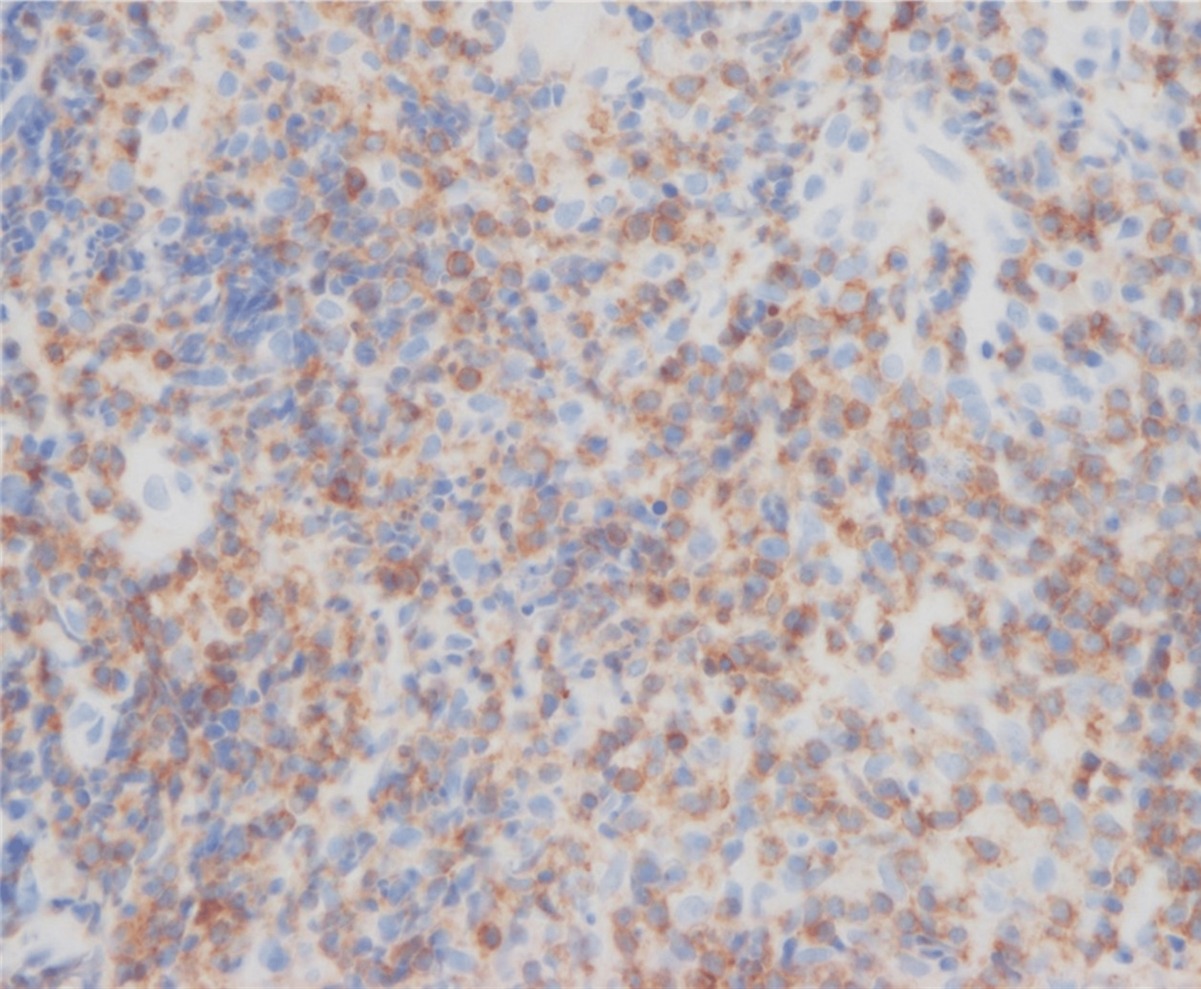
Nuclear staining for nuclear factor of activated T-cells c1 (NFATc1) in this case. In a previous study [8], the cytoplasm was stained; however, in the present case (as shown in the image), the cytoplasm is not stained.

A solitary red nodule on the nose with histologically dense lymphocytic infiltration is commonly seen. The diagnosis may be either cutaneous pseudolymphoma (CPL), lymphocytoma cutis, lymphadenosis benigna cutis, or cutaneous lymphoid hyperplasia, all of which refer to the same benign process mimicking lymphoma. CPL may occur at any age. It is not always easy to distinguish between CPL and PCSM-LPD. The latter is a better fit for our case because of the very high CD4+/CD8+ ratio, positivity of follicular helper T-cell makers, and clonal T-cell proliferation. Nuclear staining for nuclear factor of activated T-cells c1 (NFATc1) was reported to be a key finding of PCSM-LPD [8]. We performed additional staining for NFATc1. Unlike a previous study [8], NFATc1 was negative (Figure 12).

A case report of PCSM-LPD in a 33-year-old woman was reported as a young case of PCSM-LPD from Japan [9]. However, this report is considered atypical for PCSM-LPD because of the high Ki-67 (40%) and negative T cell receptor gene rearrangements [9]. Another case report from Japan is that of a 52-year-old patient [10]. He was diagnosed as PCSM-LPD, but his CD30-positive status and aggressive course also made this case atypical for PCSM-LPD [10].

## Conclusions

We experienced a young Japanese case of PCSM-LPD. This case was considered typical for PCSM-LPD, except for the fact that the patient was young. Our case suggested that young cases with PCSM-LPD may have been misdiagnosed with CPL, which may be one of the reasons why this type of lymphoproliferative disorder has been reported to occur in elderly people.

In addition, NFATc1 was not stained by either PCSM-LPD or CPL. Further investigation may be needed to determine if NFATc1 can be used to differentiate PCSM-LPD from CPL.
